# A scoping review on the use and usefulness of online symptom checkers and triage systems: How to proceed?

**DOI:** 10.3389/fmed.2022.1040926

**Published:** 2023-01-06

**Authors:** Anthony Pairon, Hilde Philips, Veronique Verhoeven

**Affiliations:** Department of Family Medicine and Population Health, University of Antwerp, Antwerp, Belgium

**Keywords:** triage, symptom checkers, diagnosis, digital health, mhealth (mobile health), ehealth

## Abstract

**Background:**

Patients are increasingly turning to the Internet for health information. Numerous online symptom checkers and digital triage tools are currently available to the general public in an effort to meet this need, simultaneously acting as a demand management strategy to aid the overburdened health care system. The implementation of these services requires an evidence-based approach, warranting a review of the available literature on this rapidly evolving topic.

**Objective:**

This scoping review aims to provide an overview of the current state of the art and identify research gaps through an analysis of the strengths and weaknesses of the presently available literature.

**Methods:**

A systematic search strategy was formed and applied to six databases: Cochrane library, NICE, DARE, NIHR, Pubmed, and Web of Science. Data extraction was performed by two researchers according to a pre-established data charting methodology allowing for a thematic analysis of the results.

**Results:**

A total of 10,250 articles were identified, and 28 publications were found eligible for inclusion. Users of these tools are often younger, female, more highly educated and technologically literate, potentially impacting digital divide and health equity. Triage algorithms remain risk-averse, which causes challenges for their accuracy. Recent evolutions in algorithms have varying degrees of success. Results on impact are highly variable, with potential effects on demand, accessibility of care, health literacy and syndromic surveillance. Both patients and healthcare providers are generally positive about the technology and seem amenable to the advice given, but there are still improvements to be made toward a more patient-centered approach. The significant heterogeneity across studies and triage systems remains the primary challenge for the field, limiting transferability of findings.

**Conclusion:**

Current evidence included in this review is characterized by significant variability in study design and outcomes, highlighting the significant challenges for future research.

An evolution toward more homogeneous methodologies, studies tailored to the intended setting, regulation and standardization of evaluations, and a patient-centered approach could benefit the field.

## 1. Introduction

Out-of-hours (OOH) medical care is currently facing an increasing demand, affecting both general practitioner cooperatives (GPCs) and emergency departments (ED) (1). This rise in workload has multifactorial origins and can be partially explained by macro-evolutions such as a population that is both growing as well as aging, thus characterized by an expanding group of care recipients with an intensifying care need per capita. In addition, a significant proportion of out-of-hours contacts are deemed medically non-urgent, constituting a tendency of unnecessary use and overdemand (1–4).

Such superfluous utilization of the available care systems could lead to an unsustainable workload. These factors further burden healthcare providers, increasing the risk of poor patient outcomes (5).

Furthermore, COVID-19 has shown that a sudden increase in healthcare seeking behavior quickly overloads a system that under normal circumstances already operates close to its maximum capacity. Additionally, a pandemic forces a minimization of face-to-face contacts adding to the need for additional pathways into the care system (1).

Overconsumption of OOH-care also comes at an increased cost, further straining the affordability of the healthcare system. Cost-effective interventions to safeguard its sustainability are therefore of primordial importance (3).

Potential demand management strategies were tested to mitigate these challenges. Co-payment, online advice, an overview of the medical cost, and a GP appointment the next morning were all investigated as measures to influence parents’ decision process in OOH-care for their children. Online advice was reported to be the only intervention that could potentially affect healthcare seeking behavior in both medically urgent and non-urgent cases without limiting the accessibility of care (6).

Online medical advice fits within a larger trend as searching the internet for health information and potential diagnoses is an increasingly common phenomenon. In Australia about 80% of the population uses the internet for health concerns and 40% searches for self-treatment advice (7). Similar results were found in the US, where about 33% of residents reports attempting to self-diagnose their symptoms via online research (8). This often serves as a precursor to a medical consultation in an attempt to assess severity and thus urgency, with the majority of UK adults consulting the internet beforehand (9).

### 1.1. Objective

Currently there are a multitude of online symptom checkers and self-triage tools available to the public. These are driven by different entities, such as government bodies with NHS 111 Online in the UK and SNS24 in Portugal, professional associations or commercial firms. However, they are often found to lack a solid evidence-based foundation concomitantly undergoing varying degrees of often self-developed validation. This scoping review aims to provide an overview of the current state of the art of this rapidly progressing field, covering user demographics, safety, accuracy, compliance, cost effectiveness, impact, user experience and complementary implementation with other demand management measures. In doing so, it endeavors to map existing limitations and gaps in the supporting evidence, providing a guide for researchers to conduct relevant studies.

## 2. Methods

The study design of our scoping review is based on Arksey and O’Malley’s (10) five-stage methodological framework.

### 2.1. Sources and search strategy

A comprehensive search strategy was developed to meet the stated objectives. It is based on three defining features that are reflected in the search terms, highlighting the necessity of having a triage and/or diagnostic function, being exclusively patient-operated, and available in a digital format. The final search strategies can be found in Supplementary Appendix 1.

It was subsequently applied to six databases: Cochrane library, NICE, DARE, NIHR, Pubmed, and Web of Science.

### 2.2. Selection criteria

The search strategy was not filtered on publication date or language, thus encompassing all published works up until July 15, 2022. Studies had to be conducted within a developed health care setting.

Guidelines, primary studies reporting an outcome, and literature reviews were considered for inclusion. Conference abstracts, presentations, opinion pieces, editorials, and comments were excluded. Studies suffering from conflicts of interest were also discarded.

This scoping review aims to cover current evidence on patient-facing digital tools that are text-based and cover a broad range of complaints and medical conditions. Thus, algorithms were excluded if they catered to health professionals, only applied to specific conditions (e.g., COVID-19), focussed on specific situations (e.g., disaster triage). Systems that required additional intervention, such as pictures, video-calls, and any other variation of teleconsultation were also excluded.

### 2.3. Study selection

Articles were screened on title and abstract after elimination of duplicates, keeping the defining characteristics in mind. Subsequently, two researchers independently performed full text evaluations of the articles considered eligible in the first stage. Periodic discussions were held throughout the review process to reach conformity in case of discordance. Additionally, the references of selected articles were screened.

### 2.4. Data charting and data extraction

Two authors performed data extraction and charting in duplicate in accordance with a predetermined protocol. The following variables were extracted: author, country, publication date, type of study, methodology, sample size, outcomes assessed, and major findings.

## 3. Results

**FIGURE 1:**
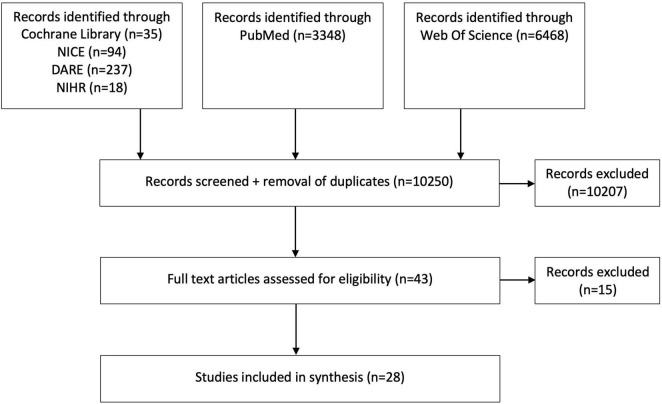
Study selection flow diagram.

A total of 10,250 articles were identified through the initial search strategy. Screening of title and abstract excluded 10,207 articles. Full text review was carried out for 43 articles. 28 studies conformed to the eligibility criteria and were included in the literature review. This process is illustrated in Figure 1.

The retained studies originated from the UK (6), the USA (6), Canada (3), the Netherlands (3), Australia (2), (Germany (2), Finland (1), Hong Kong SAR, China (1), New-Zealand (1) Norway (1), Russia (1), and Thailand (1).

Primary research comprised five cross-sectional surveys, five mixed method studies, five audit studies, one prospective cohort study, one retrospective cohort study, one retrospective observational study, one survey study, one population-based descriptive study, one user study, one critical analysis, and one retrospective analysis. The literature reviews that were withheld consisted of two systematic reviews, two scoping reviews, and one literature review. Table 1 provides a chronological overview of the included studies and the different topics they cover.

**TABLE 1:** Included studies of online symptom checkers and triage tools ordered chronologically.

Article information					Topics								
References	Date	Country	Type	Sample size (*n*)	User demo-graphic	Safety	Diagnostic accuracy	Triage accuracy	Compliance	Impact	User experience	Cost effectiveness	Combination of demand management strategies
Schmieding et al. (21)	May 2022	Germany	Follow-up audit study	22 symptom checkers, 45 case vignettes		X		X					
Kujala et al. (36)	May 2022	Finland	Cross-sectional survey	639 health care professionals							X		
Tsai et al. (35)	March 2022	USA	Mixed method study: interview study and user study	Interviews *n* = 25 Users *n* = 20							X		
Dickson et al. (17)	February 2022	UK	Retrospective cohort study	25.333 self-assessments				X					
Chan et al. (19)	December 2021	Canada	Prospective cohort study	281 hospital patients, 300 clinic patients		X		X		X			
Turner et al. (11)	November 2021	UK	Mixed method study	Qualitative survey user questionnaire: Telephone *n* = 795. Online *n* = 3.728. Interview *n* = 32. Staff interview *n* = 16.	X				X	X	X	X	X
Schmieding et al. (21)	July 2021	UK	Observational audit study	12 symptom checkers, 50 case vignettes		X	X	X		X			
Yu et al. (25)	March 2021	Germany	Survey study	15 symptom checkers, 45 case vignettes, 91 users				X					
You et al. (34)	January 2021	USA	Mixed method study	10 semi-structured interviews, 2000 user reviews							X		
Aboueid et al. (13)	January 2021	Canada	Qualitative cross-sectional survey	Survey *n* = 1.547	X						X		
Cross et al. (23)	January 2021	Australia	User study	512 simulated self-assessments							X		
Morse et al. (1)	November 2020	USA	Population-based descriptive study	26.646 self-assessments	X			X					
Schmieding et al. (24)	July 2020	Hong Kong SAR, China	Audit study	2 symptom checkers, 100 ED patient records				X					X
Hill et al. (22)	June 2020	Australia	Follow-up audit study	1.170 diagnosis vignette tests, 688 triage vignette tests			X	X		X			
Gottliebsen et al. (15)	May 2020	Norway and Sweden	Literature review	17 publications		X	X	X		X			
Sutham et al. (26)	April 2020	Thailand	Mixed method study	12 emergency physicians				X			X		X
Donovan et al. (31)	February 2020	UK	Systematic review	3 publications		X		X		X	X		
Meyer et al. (27)	January 2020	USA	Cross-sectional survey	Survey *n* = 329	X			X		X	X		
Chambers et al. (12)	August 2019	UK	Systematic review	29 publications	X	X	X	X	X	X	X	X	
Aboueid et al. (14)	May 2019	Canada	Scoping review	19 publications	X		X						
Millenson et al. (18)	September 2018	USA	Scoping review	30 publications		X							
Verzantvoort et al. (3)	June 2018	Netherlands	A prospective, cross-sectional study	Questionnaire: online *n* = 4.456 Phone *n* = 126	X		X	X	X	X	X		
Polynskaya et al. (37)	June 2018	Russia	Mixed method study	Survey *n* = 200 Interview *n* = 40 Focus group = 1							X		
Giesen et al. (6)	July 2017	Netherlands	Cross-sectional survey	Survey *n* = 377, 1.367 cases						X			
Elliot et al. (32)	December 2015	UK	Retrospective observational study	3.37 million self-assessments						X			
Semigran et al. (16)	May 2015	USA	Audit study	23 symptom checkers, 45 case vignettes		X	X	X					
Lupton et al. (29)	May 2015	New Zealand	Critical analysis	35 symptom checkers						X			
Nijland et al. (28)	2010	Netherlands	Retrospective analysis	6.538 self-assessments Survey *n* = 192	X				X				

Topics marked with “X” are covered by the corresponding study.

### 3.1. Study outcomes

#### 3.1.1. Demographics

Online symptom checkers and similar services generally have a diverse user base. However, participants are more likely to be younger, female (3, 11) and more highly educated (12). Other factors, such as having low health literacy (irrespective of education level), high technology literacy (13), or limited access to care (14) also seem to increase the likelihood of using a symptom checker. Interestingly, evidence suggests that having a condition that is perceived as stigmatizing, awkward or sensitive contributes to use of these tools (3, 13, 14), indicating that such systems could potentially lower the threshold to seek care for more intimate medical problems.

#### 3.1.2. Safety

Digital triage services are generally considered risk averse (15–17), favoring sensitivity over specificity, often over-triaging, assigning a higher urgency level than is required. A systematic review by Chambers et al. (12) concluded that the available studies reported no evidence of a detrimental effect on patient safety in both simulated and real settings. Important to note is that the strength of evidence is considered weak and thus insufficient, in part due to the limited number of adverse effects reported (18).

More recent studies note a general evolution toward less risk averse triage behavior in symptom checkers, in an effort to offer more suitable advice in low-acuity cases. However, this currently seems to impact safety, with a decreased sensitivity toward more urgent conditions and consequently more missed emergencies (19–21). One study building in part on the foundational research by Semigran et al. (16) finds an average of >40% of emergencies undertriaged by a collection of 22 systems (21).

#### 3.1.3. Accuracy

The evaluation of accuracy should be divided into two categories. Diagnostic accuracy pertains to digital services that provide a list of potential diagnoses ranked by likelihood and conformity to the clinical picture. Triage accuracy gages the precision of assigned urgency levels by these tools.

Triage accuracy may be the more significant metric, supporting the notion that triage should be the primary function of these tools. Making sure people seek appropriate care can contribute more to their health than attempting to identify the specific origin of their care need (16, 22, 23). A sentiment corroborated by patients, believing self-triage to be more useful to them (13).

##### 3.1.3.1. Diagnostic accuracy

A landmark study by Semigran et al. (16) in 2015 examining 23 symptom checkers found overall diagnostic accuracy to be lacking, with a correct primary diagnosis in only 34% of cases and within the top 20 suggested diagnoses in 58% of cases. Subsequent studies and literature reviews echoed the generally poor diagnostic performance of these algorithms and the sparse evidence surrounding it (12, 15, 20).

Furthermore, accuracy varied widely depending on the platform, setting, user and disease tested (14, 15). Performance is notably better for common conditions than rare diagnoses. Moreover, women and more highly educated users appear to be more successful at selecting their condition out of the line-up of probable diagnoses (14).

##### 3.1.3.2. Triage accuracy

Results on the accuracy of digital triage tools were more mixed. Yet, on average, these algorithms performed sub-optimally, owing in part to their risk averse nature (20, 24, 25). Even throughout the past years there generally has not been a markable improvement in signposting accuracy of these algorithms. More so, there is evidence that digital triage tool performance increasingly likens the triage decisions of a layperson, including their mistakes (21).

Important to note, however, is the significant variability in triage accuracy between the different tools. A comparative study reported values ranging from 33% to 78%, yet did not manage to reveal a decisively valid tool (16). Even though the field generally appears to be inadequate and seems to lack the capacity to evolve, some symptom checkers offer more promising results, portraying superior sensitivity, specificity, and accuracy (3, 19–21, 24, 26), even advancing in their capabilities over time (20). Such tools have the potential to effectively contribute to patients’ healthcare seeking behavior in some cases (20, 21, 24).

Certain characteristics of triage systems were found to be beneficiary to triage accuracy. A review of 36 symptom checkers and triage tools in Australia by Hill et al. (22) concluded that triage tools that take into account demographic data were more accurate than their counterparts. The use of AI algorithms also appeared to benefit performance. Furthermore Verzantvoort et al. (3) reported that tools developed by physician organizations showed above average results, as opposed to those created by a commercial entity or a government.

When examining these results, it remains primordial to consider the limited comparability across studies and systems due to significant heterogeneity of study designs, interventions, and measured endpoints (1, 12, 27). Moreover, the majority of algorithms was examined using clinical vignettes, limiting the validity as well as transferability to the real-world setting (15, 16, 21, 24).

#### 3.1.4. Compliance

There is very limited evidence available on compliance of patients that utilize digital triage tools (12, 16). Overall, patients seemed relatively inclined to follow the proposed advice, with studies finding 57–67,5% of participants to be compliant (3, 11, 28). Although users of NHS111 Online were less likely to comply than those that utilized telephone triage (67.5% vs. 88%; *p* < 0.001).

It has been reported that people were more motivated to initially seek primary care or self-management instructions after being advised to contact the emergency number or visit the ED (28), often because these recommendations were perceived as inappropriate and unnecessary owing to the risk averse nature of the algorithms (11).

In a study by Verzantvoort et al. (3) 65% of patients reported that they intended to comply with the outcome of that specific triage tool. People were more likely to follow the advice when urged to contact their own GP during office hours (75%), followed by self-care advice (67%), OOH-care (61%) and wait-and-see instructions (56%). Certain patient characteristics correlated with a higher compliance, such as <13 years (OR 1.8, 95% CI: 1.3–2.3, *p* < 0.001), male sex (OR 1.2, 95% CI: 1.1–1.4, *p* = 0.045), and user satisfaction (OR 2.5, 95% CI: 2.2–2.9, *p* < 0.001). Main reasons for defying the advice given related to inability to accurately convey complaints, contact with a physician pre-dating triage and preferring their own judgment. An important limitation of this study lies in the fact that it only quantifies intention to comply, instead of the resulting care seeking behavior (3).

#### 3.1.5. Impact

##### 3.1.5.1. Impact on health literacy

Availability of online symptom checkers and triage tools can be a valid source of health information, allowing patients to educate themselves (29) and gain further insights in their health status and conditions (27). An increase in health literacy could subsequently benefit the patient-physician relationship (22).

##### 3.1.5.2. Impact on health equity and digital divide

As established, demographics of users of symptom checkers and triage tools tend to show a younger, more educated user base. Older patients, or those less educated utilized telephone triage and direct contact more often. This could potentially affect health equity (12). In contrast, a study by Morse et al. (1) on use characteristics of a digital symptom checker found a significant proportion of patients to be of older age, belonging to a subpopulation not typically associated with regular use of online resources. These mixed results underline the need to evaluate a tool in its intended setting and population.

##### 3.1.5.3. Impact on OOH-care

Available research on the impact of triage systems on OOH-care is scarce.

###### 3.1.5.3.1. Healthcare seeking behavior

It has been reported that digital triage tools and symptom checkers have a limited ability to modify health care seeking behavior by informing patients and assisting them to make medically appropriate decisions. It was found that such guidance could potentially improve the safety of parent’s decisions in the management of children with possibly severe ailments (6). However, research was often focussed on specific conditions or settings and offered indirect evidence, limiting the ecological validity of their conclusions (12).

###### 3.1.5.3.2. Workload

Furthermore, evaluation of the impact of triage algorithms on the burden of the health care systems shows highly variable results. Some studies report a potential decrease in pressure on the health care system (3, 30). Which could, by extension, reduce the urgency of current health care staffing shortages (1).

Others, however, did not observe an effect on workload, as Donovan et al. (31) were unable to discern a digital intervention capable of altering urgent care usage, based on available data.

More importantly, the majority of studies that reported on this topic has voiced concerns that symptom checkers and triage tools might conversely increase inappropriate OOH-care use due to their risk-averse nature, often advising additional health care interaction, even though self-care or a wait and see policy would be adequate (11, 15, 20).

##### 3.1.5.4. Accessibility to care

It is relevant to note, however, that a growing demand and rise in health care utilization does not necessarily equate to an increase in inappropriate use. Implementation of NHS 111 online resulted in a significant new demand, with people finding their way to the available health care providers more easily. This suggests that symptom checkers could increase accessibility to care, lowering the threshold for those in need (11, 22). More so, people were found to be more motivated to seek medical care when assisted in their decision by a tool (12).

The inconsistent picture these studies paint of the impact of available systems on OOH-care, highlights the importance of studying a tool in its intended real-life setting to accurately assess its influence. Additionally, it will be necessary to monitor a potential shift to more appropriate use, with people previously unaware of their need of care finding their way to OOH-care services more easily, thereby likely clouding certain established outcome parameters such as workload (11, 22).

##### 3.1.5.5. Impact on public health

The data collected by such tools could also impact public health management by providing direct epidemiological data that can be used to map the evolution of infectious diseases (32). Online data generated by symptom checkers was found to capture evolutions earlier than traditional surveillance or telephone triage could. Additionally, it is able to provide insights on symptomatic patients that do not contact the health care system directly. Thus, diagnostic and triage algorithms have the potential to serve as a complementary source within national surveillance systems, especially during crises such as the COVID-19 pandemic (33).

#### 3.1.6. Cost effectiveness

There is very limited evidence available on the effect of symptom checkers and triage tools on costs. In an evaluation of NHS 111 Online during the implementation phase, costs were lower compared to NHS 111 telephone triage, potentially in part due to the on average lower acuity of complaints processed. When both systems operated simultaneously, a shift of ≥38% of telephone contacts to digital triage would be necessary to achieve a cost reduction (11).

Two small studies reported significant cost efficiency both in operational expenses as well as in care diversion. However, these savings were self-reported and considered inadequate to come to a consensus (12).

#### 3.1.7. User experience

##### 3.1.7.1. Patients

A systematic review by Chambers et al. in 2019 retained 9 studies investigating patient and/or caregiver satisfaction. Patients predominantly considered the examined symptom checkers and triage systems to be very satisfactory (12). This sentiment was corroborated by Meyer et al. (27) in their analysis of patient perspectives on the usefulness of these services. They reported patients finding the tool easy to use and useful, and made mention of a general willingness to use the tool on a recurring basis (27).

More recent research nuances this general trend to a degree. NHS 111 users were found to be more satisfied with the telephone service than the online equivalent (50% vs. 71%; *p* < 0.001) (11). Other studies showed that some prefer traditional search engines over set algorithms, mostly due to the perception that they offer more freedom in describing their symptoms and providing information (13, 23).

Several factors were observed as beneficial to usage, such as a limited accessibility to care, backing of these tools by credible sources, such as government entities and caregiver associations, or integration in the care system. Conversely, there are still multiple obstacles such as restricted internet access, the use of medical jargon, as well as reservations about data privacy and trust (13).

To overcome these hurdles and improve user experiences, several advancements have been suggested. On the one hand, a more customizable input would increase the perceived flexibility of the system, allowing patients to describe their pattern of symptoms more accurately and thus feel more heard. On the other hand, tools should be made more comprehensible and allow the patient insight into the decision-making process (34). This can be achieved by using unambiguous language and offering comprehensive explanations (13, 34, 35).

Interestingly, one study observed a significant lack of awareness of symptom checkers, with >50% of participants unfamiliar with the technology, which severely limits usage. To optimize the use of these tools, targeted interventions could be implemented, tailored to individual subpopulations of potential users (13). Furthermore, support and recommendation by health care providers and credible associations has the potential to impact use considerably (13, 35, 36).

##### 3.1.7.2. Caregivers

Evidence on the experiences of healthcare providers with symptom checkers is sparser. Current studies report a generally positive perception of the algorithms, with health care providers believing it could be beneficial to both patients, as well as caregivers (36).

Anticipated benefits relate to a potential reduction in workload, while simultaneously offering a more expeditious, accessible and supportive service to patients. Triage services were believed to be especially useful during the pandemic, when demand increased significantly (36).

Several challenges were acknowledged pertaining to the suboptimal impact on workflow with risk of multiple channels of contact per patient, the inaccuracy of triage, the effect on the digital divide, and a perceived threat to professional autonomy (36). Even so, digital diagnostics are considered a part of the future of medicine by many caregivers (37).

User experience is naturally specific to any individual tool. The overall trend in the above-mentioned reports, however, is more consistent and expected to be more generalizable (12).

#### 3.1.8. Combination of demand management measures

The implementation of an online self-triage service is one possible measure to counter the current challenges facing OOH-care systems. There is some evidence on the effects of combining these tools with other interventions, such as telephone triage.

Multiple studies reported that performance of algorithms was comparable to that of existing telephone triage services, such as NHS111 (19, 32). Parallel operation of both systems did not lead to significant reductions in telephone contacts during the initial phase, as some interacted with both, to confirm their findings (11). There are, however, indications that implementation of triage services as a complementary service to telephone triage could be beneficial to the healthcare system, as it could lower the threshold for care and offer a suitable alternative approach for lower acuity and non-trauma problems (11, 25, 26); a potential advantage echoed by telephone staff of NHS111 (11).

## 4. Discussion

A 2019 systematic review by Chambers et al. concluded that the research available at that time was considered weak, the majority consisting of observational studies, and clouded by an abundance of gray literature (12). In recent years, there has been a notable increase in research surrounding the technology as well as ehealth in general. However, there are still several limitations, both pertaining to the available tools, as well as the studies evaluating them.

Research examining safety and accuracy of these systems highlights the persistence of a risk-averse disposition (12, 15–17). An effort is being made to evolve to more balanced algorithms, but some studies report that this evolution currently comes at the expense of the technology’s safety, which should always remain their priority (19–21). More so, these developments seem to miss their mark at present, with most systems still appearing to be insufficiently accurate and show limited progress to date.

The applied methodologies to evaluate these parameters are suboptimal as well, with most of the available studies relying solely on clinical vignettes. Such simulations are a good technique to benchmark and compare different tools, offering an initial insight in their performance. They are, however, insufficient to determine functionality in a real-life setting and should serve exclusively as a basis to be supplemented by studies in the intended environment. Current research fitting these requirements was often limited by a relatively small study sample and short duration, or restricted to certain conditions and subpopulations. A finding that echoes the results of previous studies (12, 15, 34).

Future research should be implemented in the real world and be of a larger scale and scope, allowing for continuous, multifaceted data collection to monitor foundational aspects, such as safety and accuracy, and compare them against the gold standard of a medical evaluation. This would require access to personal medical information, something research groups often lacked (1, 27).

The same applies to other case-specific outcomes such as cost-effectiveness, a topic that currently remains unclear, with a handful of self-reported and non-peer reviewed studies being regarded as inadequate. An initial evaluation of NHS 111 Online did show a potential economic benefit if enough patients shifted from telephone to online triage (11). This evidence is not transferable due to the significant multifactorial influences making the results highly specific to the circumstances.

We concur with the statement that triage should be the most prominent area in the future of these systems, as it has the most potential to have a significant impact on both individual patients, and the health care system (16, 23).

On a positive note, there is more consensus regarding the experiences of users and health care professionals. Patients generally found the tool user-friendly, usable, and useful (12, 27).

Healthcare professionals too believe in their potential benefit to both patients and caregivers (36) and reportedly envision a role for it in the future of medicine (37). There are, however, still some hurdles to overcome, such as the use of medical jargon and questions surrounding privacy, which hinder trust (13). These findings are expected to be more generalizable to different tools and settings (12).

To further optimize patient experiences, tools should become increasingly more patient-centered. This can be achieved through a transparent policy, understandable language and comprehensive explanations (13, 34, 35), thus further empowering patients and enhancing trust. Ideally, patients should be included in the design process of these tools.

Evidence on compliance was scarcer, finding users to be relatively inclined to follow the guidance given (3, 11, 28), but often apprehensive to follow through because of advice perceived as excessive owing to the risk-averse reactions of most tools (11, 28). The above-mentioned research mostly measured intention to comply, rather than real behavior, severely limiting the strength of evidence. Compliance should therefore be investigated by tracking patient flow and measuring healthcare seeking habits in a real life setting. In addition, the impact of factors influencing compliance, should be mapped.

The impact of tools on the workload remains uncertain, with highly variable results being reported. The top-performing apps have the potential to influence healthcare seeking behavior toward appropriate care, with both patients and the healthcare system benefitting (3, 6, 30). However, multiple studies demonstrated how the risk-averse disposition of some tools could conversely lead to an increase in healthcare demand, thus foregoing its value in this regard (11, 15, 20).

Evidence suggests, however, that their role reaches further than demand management. Digital triage tools could open a new avenue to connect with the healthcare system, lowering the threshold to some and improving the accessibility of care (11, 22). Moreover, with patients increasingly turning toward the internet for health information, there is a clear need for validated information, which symptom checkers could provide through tailored advice, potentially positively impacting health literacy in the process (6, 12). Conversely, concerns have been voiced that preliminary self-diagnosis through these systems could contribute to increased anxiety in some patients (16). These conflicting statements warrant additional research on patient perspectives.

There is some concern about the impact of this technology on the existing digital divide and the potential consequences for health equity. Current research reports a user base skewed more toward those younger, more educated and technologically literate (3, 11–13), potentially posing a challenge for certain subpopulations, such as the elderly. Implementation of these tools should therefore be as an adjunct to other channels, rather than as a replacement (11, 25, 26).

Additionally, a potential advantage of these tools in crises was illustrated by their use in the pandemic. A veritable plethora of tools was created within a short time frame to help curb the exponential demand in healthcare seeking, as became apparent during our search strategy. They served a double function, unburdening the healthcare system by providing trustworthy background information and guidance in times of an information overflow (36), as well as collecting epidemiological data for national surveillance (33).

We therefore recommend that clear objectives be set prior to implementing and researching the tools, allowing for relevant and appropriate outcomes to be studied. This approach facilitates a more comprehensive understanding of the impact on the healthcare system, including the effects on demand management, accessibility of care and patient education. It is this all-encompassing analysis that can determine the ultimate added value of the technology.

The primary challenge for the field remains the significant heterogeneity across studies and triage systems. The tools were shown to vary significantly in terms of functionality, performance, and triage approach. The additional variety in study designs, interventions, quality and measured endpoints of current literature further limits the generalizability of results. This observation is a confirmation of previous findings, underlining the persistence of this shortcoming (12, 14, 18–20). Algorithms will need to be studied in their intended environment to draw more definitive conclusions.

There are several risks to the field as well, with a multitude of tools flooding a largely unregulated market, often lacking an adequate evidence-based approach and suffering from conflicts of interest. This could be amended by providing independent and transparent research to assist patients and caregivers in identifying the top performing systems (35). Implementation should be conducted within a framework of standardized evaluations to objectively validate triage systems, contributing to regulation of the field (15, 17, 18, 20). Responsible authorities should subsequently provide oversight, recommend validated tools and integrate them into the health care system to optimize functionality and user experience (13, 36).

### 4.1. Limitations of this review

There are several limitations to this review. It is possible that some potentially eligible articles were not captured by our search method. This could be due to the selection of databases, the applied exclusion criteria or the selected search terms which highlighted a lack of applicable MeSH terms. Furthermore, the absence of a formal quality assessment of the retained studies can make it challenging to accurately appraise the value of reported results to the field.

## 5. Conclusion

Numerous digital symptom checkers and triage tools are presently available to the public and fit within the trend of an increasing reliance on the internet for access to health information.

The evidence collected in this literature study is characterized by multiple limitations. Nevertheless, with some reservation, several trends can be distilled. Current research highlights the risk-averse nature of these services, which causes challenges for their accuracy. Recent evolutions in algorithms have varying degrees of success.

User satisfaction is generally high, and patients appear to be amenable to the advice given by a digital service, with most participants intending to comply. There is evidence of a multifaceted impact on the healthcare system, with preliminary research seeing potential benefits for accessibility of care, health literacy and syndromic surveillance. In contrast, there is ambiguity about the effects on workload and digital divide, warranting caution.

Notwithstanding these themes, there is a clear need for additional research, with a strong preference for study designs that most closely match the circumstances of the intended definitive setting. Additionally, an evolution toward more homogeneous methodologies, aided by regulation and standardization of evaluations, should increase the generalizability of results, furthering the field as a whole.

## Author contributions

AP, HP, and VV designed the review collectively and were responsible for data interpretation and reporting. AP and HP were involved in planning. AP and VV conducted the review. AP attested that all listed authors met authorship criteria and that no others meeting the criteria have been omitted. All authors contributed to the article and approved the submitted version.
