# Supplementary material for: A scoping review on the use and usefulness of online symptom checkers and triage systems: How to proceed?
Source: Front Med (Lausanne). 2023 Jan 6;9:1040926. doi: 10.3389/fmed.2022.1040926 (PMC9853165; doi:10.3389/fmed.2022.1040926)
Supplement: Supplementary file 1 [file Data_Sheet_1.DOCX]

Supplementary Appendix 1: Database search strategies

**Database: Cochrane library –** Last accessed on 08/07/2022

**--------------------------------------------------------------------------------**

1. (“triage”) (35)

**Database: NICE –** Last accessed on 08/07/2022

**--------------------------------------------------------------------------------**

1. (“triage”) (94)

**Database: DARE –** Last accessed on 08/07/2022

**--------------------------------------------------------------------------------**

1. (“triage”) (237)

**Database: NIHR –** Last accessed on 08/07/2022

**--------------------------------------------------------------------------------**

1. (“triage”) (18)

**Database: PubMed –** Last accessed on 08/07/2022

**--------------------------------------------------------------------**

1. (“triage service”) OR (“symptom*checker*”) OR (triage[MeSH Terms]) (14.497)
2. (“self diagnosis”) OR (“self referral”) OR (“self triage”) (2.301)
3. (#1) OR (#2) (16.736)
4. (online) OR (digital) OR (mobile) OR (internet) OR (computer) OR (smartphone) OR (electronic) (4.439.056)
5. (#3) AND (#4) (3.794)
6. (“mass casualty”) OR (disaster) (122.060)
7. (#5) NOT (#6) (3.348)

**Database: Web of Science –** Last accessed on 08/07/2022

**-----------------------------------------------------------------------------**

1. (TS=(Triage service) OR TS=(Symptom*checker) OR TS=(Triage) OR TS=(“self diagnosis”) OR TS=(“self referral”) OR TS=(“self triage”)) (40.709)
2. (TS=(online) OR TS=(digital) OR TS=(mobile) OR TS=(internet) OR TS=(computer) OR TS=(smartphone) OR TS=(electronic)) (8.335.722)
3. (TS=(“mass casualty”) OR TS=(disaster)) (211.936)
4. #1 AND #2 NOT #3 (6.486)
